# Identification of pAKT as a pharmacodynamic marker for MER kinase in human melanoma G361 cells

**DOI:** 10.1186/s40364-020-0184-9

**Published:** 2020-02-04

**Authors:** Yaoyu Chen, Margaret Favata, Michelle Pusey, Jun Li, Yvonne Lo, Min Ye, Richard Wynn, Xiaozhao Wang, Wenqing Yao, Yingnan Chen

**Affiliations:** Incyte Research Institute, 1801 Augustine Cut-off, Wilmington, DE 19803 USA

**Keywords:** Biomarker, MER kinase, Phosphorylated AKT

## Abstract

**Background:**

The MER signaling pathway represents an attractive therapeutic target for human cancers. Growth arrest–specific protein 6 (GAS6)–induced MER phosphorylation is often unstable and difficult to detect without pervanadate pretreatment in human cancer cells, posing a challenge for the development of selective MER kinase inhibitors. Here, we identified phosphorylated AKT (pAKT) as a specific pharmacodynamic marker for MER kinase inhibitors in human melanoma G361 cells.

**Methods:**

The expression of MER, TYRO3, and AXL were profiled among multiple human cancer cells. To determine whether they play a role in the activation of pAKT, MER and TYRO3 were selectively depleted by small, interfering RNA knockdown. In addition, using AKT phosphorylation as a readout, a high-throughput cell-based assay was established in G361 cells for evaluation of the potency of potential inhibitors of MER pathway activation.

**Results:**

We demonstrated that high levels of MER and TYRO3, but not AXL, were expressed in G361 cells. In these cells, pAKT was induced by GAS6 treatment, which could be reversed by AXL/MER inhibitors. We showed that GAS6-induced pAKT is only dependent on MER kinase, but not TYRO3, in G361 cells. Furthermore, we observed a correlation in potency between inhibition of pAKT in G361 cells and pMER in MER-overexpressing Ba/F3 cells by these inhibitors.

**Conclusions:**

In summary, we have demonstrated that GAS6-induced pAKT is a possible pharmacodynamic marker for the inhibition of MER kinase, and we have successfully developed a cell-based functional assay for screening small-molecule inhibitors of MER kinase for potential therapeutic utility in treating GAS6/MER-deregulated human cancers.

## Introduction

Abnormalities in receptor tyrosine kinases are involved in a wide range of important biological activities in cancer, including cell proliferation, differentiation, and drug resistance [1]. The TAM family of receptor tyrosine kinases includes 3 members: TYRO3, AXL, and MER [2]. Binding of ligands to the these receptors was reported to lead to activation of the TAM kinases, as well as PI3K/AKT, RAS/RAF/MAPK, and JAK/STAT signaling pathways in human cancer cells [2–4]. TAM activation and signaling have been involved in multiple cellular functions, including cell growth arrest, cell survival, cell proliferation, cell adhesion, and inflammation [4, 5]. In addition, TAM activation was shown to regulate innate immunity including inflammatory pathways in dendritic cells and macrophages, clearance of apoptotic cells, and differentiation of natural killer cells [2–8].

Among the 3 TAM family members, AXL has been an attractive target for the treatment of cancer [9, 10]. Both small-molecule AXL kinase inhibitors and biological reagents that block the interaction between growth arrest–specific protein 6 (GAS6) and AXL have been reported at various stages of drug development [11]. Stimulation of melanoma cells with GAS6 has been reported to activate several downstream signaling pathways including MAPK, AKT, and JAK/STAT. MER tyrosine kinase inhibition via small hairpin RNA reduced downstream signaling and inhibited tumor growth in an animal model, suggesting that MER kinase may be an attractive target for the treatment of human cancers [12]. MER expression has been reported in a variety of tumors, including melanoma and pre–B-cell acute lymphoblastic leukemia [5, 13, 14], and ectopic expression of *MER* in lymphocytes in transgenic mice promotes the development of leukemia/lymphoma [5, 13]. MER is also implicated in other human conditions, such as autoimmune disease and thrombosis [2].

Extensive research has been conducted to identify MER-selective small-molecule inhibitors; for example, Graham et al. reported on the MER inhibitors UNC569, UNC1063, and UNC2025 by comparing the levels of phosphorylated MER (pMER) in cancer cells treated with pervanadate [15–18]. MER phosphorylation is dependent on binding of its ligand GAS6 or protein S [19, 20]; however, ligand-activated pMER is often unstable and difficult to detect without pervanadate pretreatment in human cancer cells, impeding the development of a selective MER kinase inhibitor [18]. Therefore, it is important to identify a specific pharmacodynamic (PD) marker to monitor MER kinase activity in human cancer cells.

In this study, we profile the expression of MER, TYRO3, and AXL among multiple human cancer cells, and assess induction of phosphorylated AKT (pAKT) by GAS6 and reversal by AXL/MER inhibitors in human melanoma G361 cells that were found to express high levels of MER and TYRO3, but not AXL. We demonstrate that GAS6-induced pAKT is a possible PD marker for the inhibition of MER kinase in G361 cells, and developed a cell-based functional assay for screening small-molecule inhibitors of MER kinase for potential therapeutic utility in treating GAS6/MER-deregulated human cancers.

## Materials and methods

### Materials

HeLa, DU145, THP-1, RKO, SKM1, A549, OCI-LY3, G361, and HL60 human cancer cell lines were obtained from ATCC (Manassas, VA, USA). Roswell Park Memorial Institute (RPMI) 1640 medium, penicillin-streptomycin and 0.05% trypsin were from Gibco (Carlsbad, CA, USA). Heat-inactivated fetal bovine serum (FBS) was purchased from Hyclone (South Logan, UT, USA). Anti-pAKT (S473) #9271, anti-AXL (C44G1) #4566, anti-MER (D21F11) #4319, anti-TYRO3 (D38C6) #5585, and anti-rabbit Alexa 488 antibody were purchased from Cell Signaling Technology (Danvers, MA, USA).

### Cell culture

Human cancer cells were grown in RPMI with 10% heat-inactivated FBS plus 1% penicillin-streptomycin at 37 °C with 5% CO_2_. All human cancer cell lines were split every 3 to 4 days using 0.05% Trypsin-ethylenediaminetetraacetic acid (Trypsin-EDTA).

### siRNA

Small, interfering RNA (siRNA) reagents to knock down each individual gene were from Dharmacon (Lafayette, CO, USA). For each transfection, 30 pmol of siRNAs (a mixture of 4 different siRNAs per gene) were transfected into cells using RNAiMax (Invitrogen, Waltham, MA, USA) with 2.5 mL of growth medium according to the manufacturer’s protocol. Knockdown efficiency was examined after 72 h by Western blotting.

### TAM kinase assay

The assay buffer contained 50 mM HEPES, pH 7.5, 10 mM MgCl_2_, 1 mM ethylene glycol tetraacetic acid, 0.01% NP-40, and 2 mM dithiothreitol. Test inhibitors (0.5 μL) dissolved in dimethyl sulfoxide (DMSO; 2.5% final concentration) were transferred to white 384-well assay plates (Greiner LUMITRAC™ plates, Sigma-Aldrich, St Louis, MO, USA). Enzyme solutions of 13.8 nM AXL (Life Technologies, Waltham, MA, USA, PV4275), 4.1 nM MER (Life Technologies, PV4112), or 0.366 nM TYRO3 (Life Technologies, PR7480A) were prepared in assay buffer. A 1 mM stock solution of peptide substrate Biotin-EQEDEPEGDYFEWLE-amide (Quality Controlled Biochemicals, Hopkinton, MA, USA) dissolved in DMSO was diluted to 1 μM in assay buffer containing 100 μM ATP (for AXL and MER assays) or 20 μM ATP (for TYRO3 assay).

Next, 10 μL enzyme solution (or assay buffer for the enzyme blank) was added to the appropriate wells in each plate, and 10 μL/well substrate solution was added to initiate the reaction. The plate was protected from light and incubated at room temperature for 1 h. The reaction was stopped by adding 10 μL detection solution containing 50 mM Tris-HCl, pH 7.8, 150 mM NaCl, 0.05% bovine serum albumin (BSA), 45 mM EDTA, 180 nM streptavidin-allophycocyanin (Perkin Elmer, Waltham, MA, USA, CR130-100) and 3 nM Eu-W1024 anti-phosphotyrosine PY20 (Perkin Elmer, AD0067). The plate was incubated for 1 h at room temperature, and homogenous time-resolved fluorescence signal was measured on a PHERAstar FS plate reader (BMG LABTECH, Cary, NC, USA). Percentage inhibition was calculated for each concentration, and half-maximal inhibitory concentration (IC_50_) values were generated from curve fitting with GraphPad Prism software.

### Western blotting

Cells were lysed in radioimmune precipitation assay buffer containing halt protease and phosphatase inhibitor mixture (Thermo Scientific, Waltham, MA, USA). Lysates were spun at 16,000 g at 4 °C for 30 min and normalized for protein concentration. For Western blotting, the protein concentration was determined and the lysates adjusted so that 50 μg was loaded per lane. Total cell lysates were separated by sodium dodecyl sulfate-polyacrylamide gel electrophoresis and electrotransferred to nitrocellulose membranes (Invitrogen). Membranes were blocked in phosphate-buffered saline (PBS) plus 0.1% (v/v) Tween 20 (PBS-T) and 4% (w/v) nonfat dry milk (Bio-Rad, Hercules, CA, USA) for 1 h on a shaker at room temperature. Primary antibodies were added to the blocking solution at 1:1000 (MER, AXL, or TYRO3) and 1:10,000 (GAPDH, Cell Signaling Technology, catalog no. 2118S) dilutions and incubated and rocked overnight at 4 °C. Immunoblots were washed 3 times for 5 min each with PBS-T, and the secondary antibody was added at a 1:10,000 dilution into PBS-T milk for 1 h on a shaker at room temperature. After several washes, enhanced chemiluminescence reactions were performed according to the manufacturer’s recommendations (Supersignal West Dura extended duration substrate, Thermo Scientific).

### pAKT high-content imaging assays and data analysis

G361 cells at 2 × 10^4^ were plated in a black-walled, clear-bottom, 96-well tissue culture plate in 100 μL of growth media and incubated at 37 °C with 5% CO_2_ overnight. Cells were treated with AXL/MER inhibitors for 1 h followed by 2 μg/mL GAS6 (R&D Systems, Minneapolis, MN, USA) to activate the MER signaling pathway for 20 min, then fixed with 4% formaldehyde at room temperature for 30 min. Fixed G361 cells were permeabilized by incubating with 20 mL of permeabilization buffer (0.2% Triton X-100 in Dulbecco’s PBS [DPBS]) for 10 min at room temperature, blocked by incubation in blocking buffer (0.1% BSA in DPBS) for 30 min at room temperature, and stained with primary anti-pAKT antibody (1:300 in blocking buffer) at 4 °C overnight. G361 cells were then washed 3 times with DPBS and stained with the secondary antibody (1:1000 in blocking buffer) together with 5 mg/mL of Hoechst 33342 for 2 h at room temperature.

The Cellomics ArrayScan VTI system (Cellomics, Pittsburgh, PA, USA) was used to acquire and analyze images using the Cell Health Profiling BioApplication. The XF100-Hoechst [365(50)/535(45)] and XF100-FITC [475(40)/535(45)] filters were used with an X-Cite 120Q excitation light source to collect images of the Hoechst and fluorescein isothiocyanate (FITC) channels (Lumen Dynamics, Mississauga, ON, Canada). Images of 500 cells were captured for each well. Images in the Hoechst channel were used to identify cells with a fixed threshold. A circle was drawn with a radius of 40 pixels outside the cell border, and the total intensities of the FITC channel inside the circle were used to plot graphs. Luminescence values were used to calculate the inhibition of pAKT relative to DMSO-treated cells (0% inhibition). IC_50_ was further calculated.

### pMER ELISA assay for MER-HA overexpressing Ba/F3 cells

The cytoplasmic domain of MER fused with dimerization sequence and HA tag was cloned into a pMSCV vector with a puromycin-resistance marker to generate the construct by electroporation. Single clones that were interleukin 3–independent and puromycin-resistant were selected and characterized. Cells with stable expression of MER were selected and designated MER-overexpressing Ba/F3 cells. An R&D enzyme-linked immunosorbent assay (ELISA) plate was coated with anti-HA (H3663, 1 μg/mL, 100 μL/well) overnight at 4 °C. Ba/F3-MER cells at 1.25 × 10^5^ were treated with AXL/MER inhibitor for 1 h, before being added with 100 μL lysis buffer. Then 100 μL Ba/F3-MER cell lysis was added to the ELISA plate and incubated for 2 h. Next, 50 μL Eu-labeled anti–P-tyrosine antibodies (50 ng/well, PY-20) were added and incubated for 2 h at room temperature (with slow shaking), then the plate was washed 4 to 6 times. Finally, 200 μL DELFIA enhancement solution was added to each well and incubated for 15 min at room temperature, followed by measuring time-resolved fluorescence.

### Data analysis

For TAM kinase assays, the average values of G361 cells stimulated with 2 μg/mL GAS6 in the presence of 0.2% DMSO were used as the positive control. Unstimulated cells containing 0.2% DMSO were used as the negative control. Results were plotted as mean total intensity. The IC_50_ was defined as the concentration of test inhibitor corresponding to 50% inhibition derived from the 11-point fitted curve as determined using a 4-parameter logistic regression model. Statistical analyses were performed using GraphPad Prism (v 6.01). Student’s *t* tests were performed to statistically analyze the results, with *p* < 0.05 considered significant. All graphical data were presented as mean ± standard deviation.

## Results

### GAS6 induces pAKT in human melanoma G361 cells

**Fig. 1:**
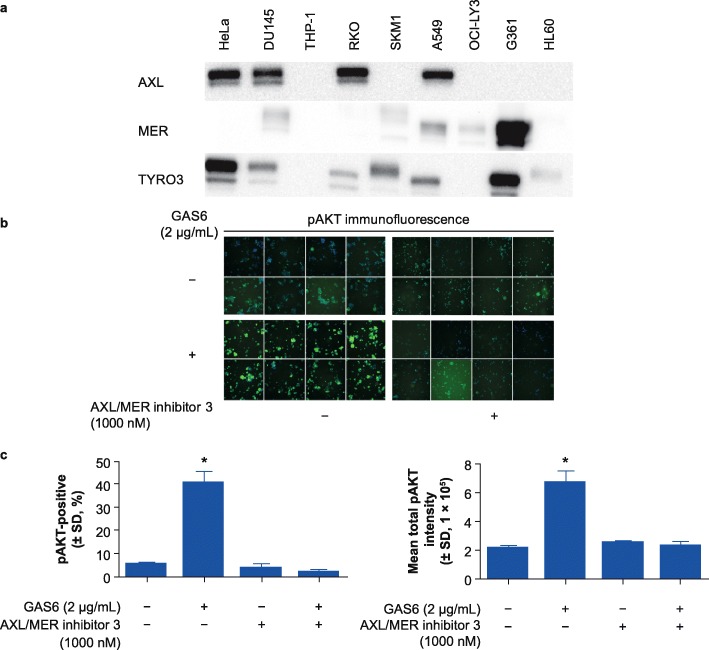
GAS6 induces pAKT in human melanoma G361 cells. **a** Western blot showing the expression of AXL, MER, and TYRO3 among 9 different human cancer cell lines. **b** and **c** High-content imaging assay showing robust phosphorylated AKT (pAKT) activation upon growth arrest–specific protein 6 (GAS6) stimulation. GAS6-activated pAKT can be reversed by AXL/MER inhibitor 3 in G361 cells. Three independent experiments were conducted. Results are shown as mean ± standard deviation (SD) of 3 wells from a representative experiment (*p < 0.05)

To identify the potential downstream component of the GAS6/MER pathway, we compared the expression of MER, AXL, and TYRO3 among various human cancer cell lines including HeLa, DU145, THP-1, RKO, SKM1, A549, OCI-LY3, G361, and HL60 (Fig. 1a). A high expression of MER and TYRO3 kinases, but not AXL, was observed in G361 cells. In contrast, other human cancer cell lines did not show a robust expression of MER kinase. Next, we tested whether GAS6 could induce significant expression of pAKT in G361 cells. Using high-content imaging analysis, a 3- to 4-fold increase of pAKT was observed after 20 min of treatment with 2 μg/mL GAS6, and this increase could be reversed by AXL/MER inhibitor (Fig. 1b and c). Therefore, G361 cells were chosen for GAS6/MER cellular assay development and PD marker validation.

### MER kinase is required for GAS6-induced pAKT in G361 cells

**Fig. 2:**
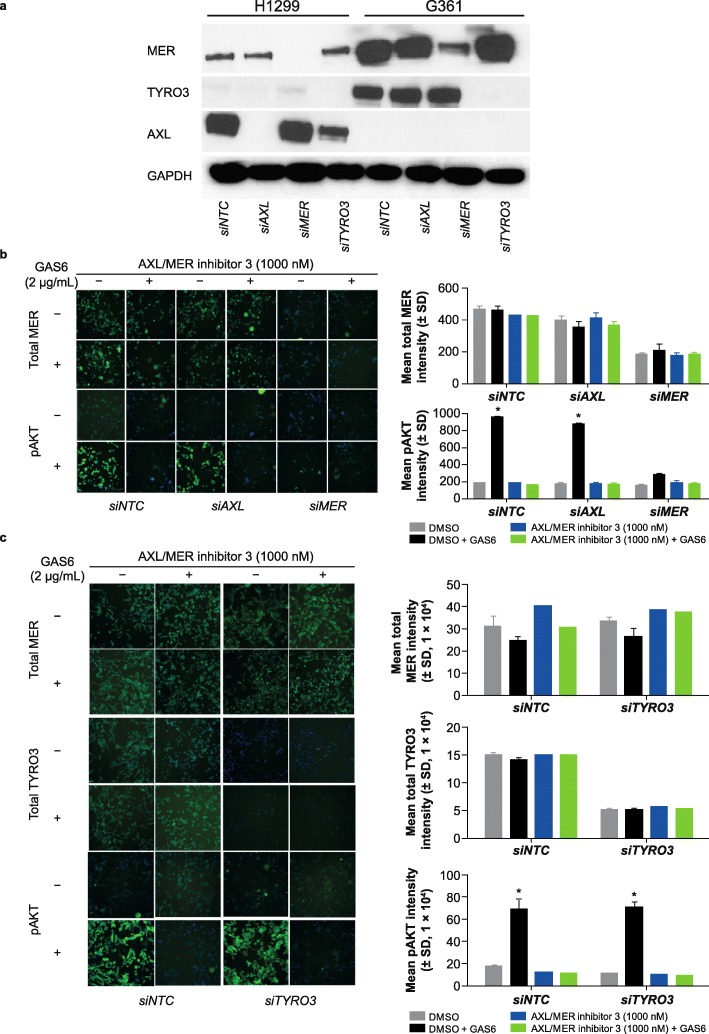
MER kinase is required for GAS6–induced pAKT in G361 cells. **a** Western blot showing knockdown of MER and TYRO3 in G361 cells. **b** Knockdown of MER fully inhibits growth arrest–specific protein 6 (GAS6)-induced phosphorylated AKT (pAKT) in G361 cells. Results are shown as mean ± standard deviation (SD) of 3 wells (*p < 0.05). **c** TYRO3 knockdown did not affect total MER and pAKT in G361 cells upon GAS6 treatment. Results are shown as mean ± SD of 3 wells (*p < 0.05). A mixture of 4 small, interfering RNAs was used per gene to knockdown AXL, MER, and TYRO3. DMSO: dimethyl sulfoxide; NTC: non-targeting control

Since G361 cells showed high expression of both MER and TYRO3 kinases, GAS6-induced pAKT could occur through either kinase. To investigate the role of MER kinase in regulating GAS6-induced pAKT, we knocked down MER and TYRO3 kinases in G361 cells. *siMER*, *siAXL,* and *siTYRO3* showed a robust reduction of MER, AXL, and TYRO3 expression in H1299 cells, and MER and TYRO3 in G361 cells (which do not express AXL kinase) (Fig. 2a). The knockdown of each gene and levels of pAKT were analyzed via high-content imaging assays. The pAKT levels in G361 cells increased 3- to 4-fold after a 15-min treatment with 2 μg/mL GAS6, which was reversed either by AXL/MER inhibitor 3 or by *siMER,* but not *siNTC* or *siAXL* (Fig. 2b). In contrast, the increase of pAKT levels was not reversed by *siTYRO3*-mediated knockdown of TYRO3 (Fig. 2c). These data demonstrate that MER kinase but not TYRO3 kinase is required for GAS6-induced pAKT in human melanoma G361 cells.

### AXL/MER inhibitors reduce GAS6-stimulated pAKT signal in a concentration-dependent manner

**Fig. 3:**
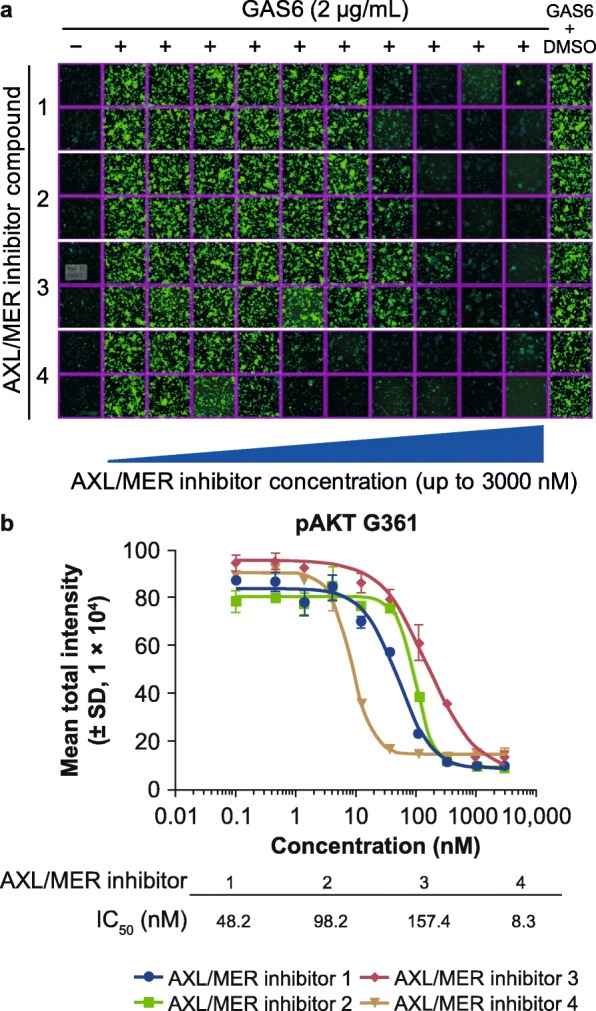
AXL/MER inhibitors reduce GAS6-stimulated pAKT signal in a concentration-dependent manner. **a** Representative images of phosphorylated AKT (pAKT) with and without growth arrest–specific protein 6 (GAS6) stimulation. pAKT in G361 cells shows dose response to AXL/MER kinase inhibitors. Green fluorescence represents cells from individual wells expressing pAKT, stained using primary anti-pAKT antibody followed by fluorescein isothiocyanate conjugated secondary antibody. **b** Nonlinear regression analysis of inhibitor concentration and pAKT response for 4 AXL/MER kinase inhibitors. Results are shown as mean ± standard deviation (SD) of 3 wells. DMSO: dimethyl sulfoxide; IC_50_: half-maximal inhibitory concentration

Given the specific role of MER kinase in G361 cells, we investigated whether GAS6-stimulated pAKT could be developed as a cellular assay. Four AXL/MER inhibitors with varied biochemical potencies (data not shown) were tested in the high-content imaging assays in the presence of GAS6. The imaging data showed AXL/MER inhibitors reduced GAS6-stimulated pAKT in a concentration-dependent manner (Fig. 3a). The IC_50_s required for pAKT inhibition from these 4 AXL/MER inhibitors were generated and shown in Fig. 3b, suggesting a potential GAS6/MER cellular assay for testing AXL/MER inhibitors.

### pAKT inhibition in GAS6-treated G361 cells may represent pMER inhibition in MER-overexpressed Ba/F3 cells

**Fig. 4:**
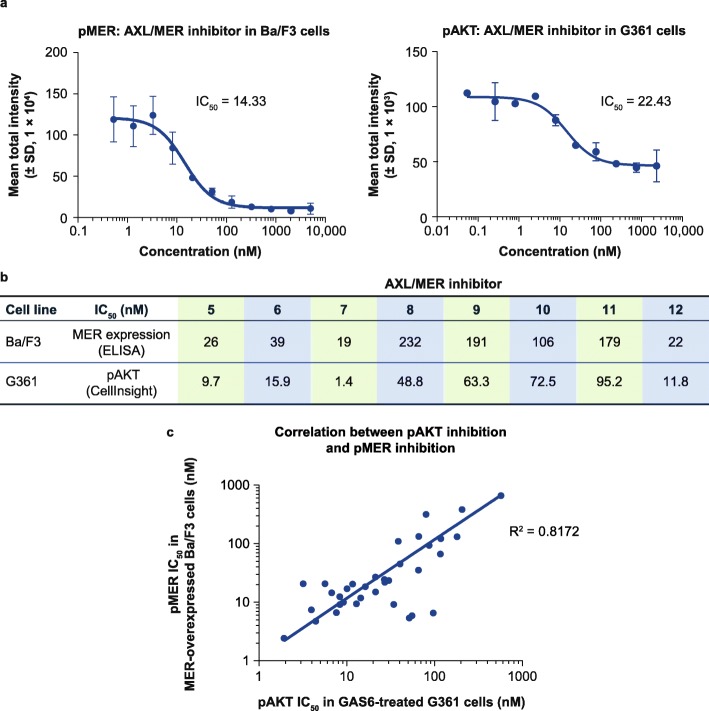
IC_50_ for pAKT inhibition in G361 cells and pMER inhibition in MER overexpressed Ba/F3 cells. **a** Nonlinear regression analysis of AXL/MER inhibitor concentration and phosphorylated MER (pMER) inhibition in MER overexpressed Ba/F3 cells (left panel), and pAKT inhibition in G361 cells (right panel). Results are shown as mean ± standard deviation (SD) of 3 wells. **b** Comparison of cellular half-maximal inhibitory concentration (IC_50_) for pMER inhibition in MER overexpressed Ba/F3 cells and pAKT inhibition in G361 cells. **c** Correlation between pAKT inhibition in growth arrest–specific protein 6 (GAS6)-treated G361 cells and pMER inhibition in MER-overexpressed Ba/F3 cells. ELISA: enzyme-linked immunosorbent assay

To further confirm whether pAKT inhibition correlates with the activity of the GAS6-stimulated MER pathway in G361 cells, we compared the potency of AXL/MER inhibitors between pAKT inhibition in GAS6-treated G361 cells and pMER inhibition in MER-overexpressed Ba/F3 cells. An example shown in Fig. 4a demonstrated that AXL/MER inhibitors reduced pAKT in GAS6-treated G361 cells with a similar trend of pMER inhibition in MER-overexpressed Ba/F3 cells. The calculated IC_50_s of 8 different AXL/MER inhibitors from these 2 assays are shown in Fig. 4b. A correlation of the 2 activities was graphed and shown in Fig. 4c. These data suggest a potential correlation between pAKT inhibition in GAS6-treated G361 cells and pMER inhibition in MER-overexpressed Ba/F3 cells.

### MER kinase selective inhibitor reduces GAS6-stimulated AKT phosphorylation in G361 but not H1299 cells

**Fig. 5:**
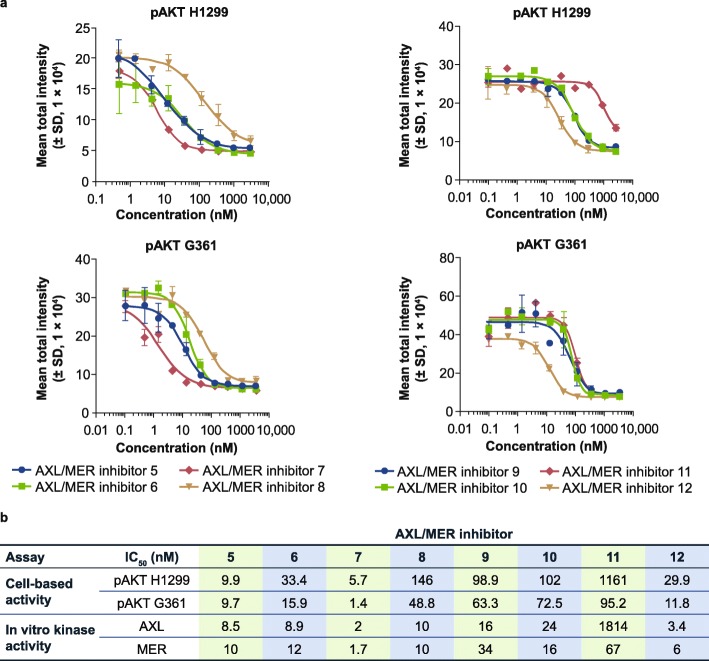
Comparison of IC_50_ for pAKT inhibition between H1299 and G361 cell lines. **a** Nonlinear regression analysis of inhibitor concentration and phosphorylated AKT (pAKT) response for 4 AXL/MER kinase inhibitors in H1299 and G361 cell lines. Results are shown as mean ± standard deviation (SD) of 3 wells. **b** Comparison of cellular half-maximal inhibitory concentration (IC_50_) for pAKT inhibition and enzymatic activities for AXL/MER

In contrast to G361 cells, H1299 cells exhibit AXL expression (Fig. 2a), and it has been shown previously that pAKT is activated by the GAS6-stimulated AXL pathway in H1299 cells [21]. To test the selectivity of the MER inhibitor, we compared pAKT inhibition between H1299 and G361 cells with AXL/MER inhibitors that showed different potencies in AXL/MER enzymatic assays. Whereas most AXL/MER inhibitors showed similar potency in pAKT inhibition between G361 cells and H1299 cells due to their dual biochemical activities (Fig. 5a and b), inhibitor 11 exhibited MER in vitro kinase inhibition with an IC_50_ of 67 nM and AXL in vitro kinase inhibition with an IC_50_ of 1814 nM. Consistent with this result, pAKT inhibition occurred at an IC_50_ of 95 nM in G361 cells, and an IC_50_ of 1161 nM in H1299 cells (Fig. 5b). These results suggest that pAKT can be induced via both AXL and MER kinases in H1299 but only MER kinase in G361 cells, and therefore may be used as models for differentiating cellular AXL and MER activity.

## Discussion

**Fig. 6:**
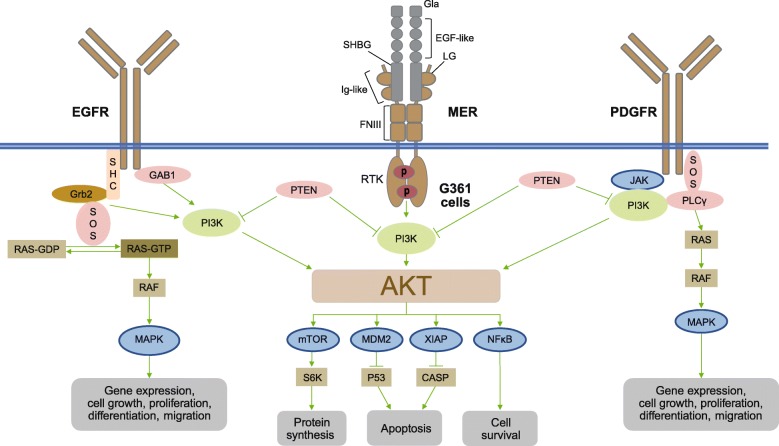
Proposed model of GAS6-inducible pAKT as a biomarker for MER kinase in G361 cells. EGF(R): epidermal growth factor receptor; FNIII: fibronectin type III; GAS6: growth arrest–specific protein 6; Gla: γ-carboxyglutamic acid; Ig: immunoglobin; LG: laminin G; PDGFR: platelet-derived growth factor receptor; RTK: receptor tyrosine kinase; SHBG: sex hormone–binding globulin

The GAS6/MER signaling pathway has emerged as a therapeutic target for human cancer and immune disorders [2, 4]. Identification of a PD marker for MER kinase and establishing a robust endogenous cellular system would be important to allow evaluation of the potency of GAS6/MER pathway inhibitors. AKT has been reported as a key downstream component in the GAS6/AXL pathway, and its phosphorylation may be used as an indication of AXL activation [21]. However, the downstream component of the GAS6/MER pathway has not been elucidated. In our study, we identified the human melanoma G361 cell line as a good model system to assay the activation of the MER signaling pathway. We profiled the expression of MER among multiple human cancer cells and found that MER and TYRO3, but not AXL, show high levels of protein expression in G361 cells. This is consistent with earlier results demonstrating that melanoma cells expressed very little AXL and relied more on the MER and TYRO3 kinases [12]. In G361 cells, pAKT is induced by GAS6 treatment and the induction of pAKT can be reversed by AXL/MER inhibitors. We also demonstrated that GAS6-induced pAKT is only dependent on MER kinase, but not TYRO3 via siRNA study. We focused not only on MER expression level in various human cancer cell lines but also on whether their GAS6-induced AKT phosphorylation was dependent on MER receptor status; this was a key factor that led to identifying G361 cells as a suitable cell line for the endogenous MER cellular assay (Fig. 6).

Recently, efforts have been made to identify MER-selective small-molecule inhibitors for human cancer [15–17]. However, one hurdle in the development of a selective MER kinase inhibitor is that ligand-activated pMER is unstable and difficult to observe without pervanadate pretreatment. For most current MER-selective inhibitors like UNC569, UNC2025, or UNC3133, their cellular activities were measured under pervanadate pretreatment in human cancer cells [15–17]. However, pervanadate pretreatment may interfere with cellular activity; hence, it is necessary to identify a PD marker for MER kinase and establish a robust endogenous cellular system to evaluate MER kinase activity. We identified that GAS6-induced AKT phosphorylation is dependent on the MER receptor in G361 cells and further used the high-throughput high-content imaging system to evaluate compound inhibitor potency in inhibiting MER pathway activation.

pAKT is an important biomarker for human cancer [22–25]. To establish pAKT as a direct phosphorylation target of MER kinase, we developed a MER cellular assay and compared the inhibition of MER activation and AKT phosphorylation. As endogenous pMER is difficult to detect, we measured the pMER readout in MER-overexpressed Ba/F3 cells. We tested 8 different AXL/MER inhibitors and were able to demonstrate correlation between pAKT inhibition in GAS6-stimulated G361 cells and pMER inhibition in MER-overexpressed Ba/F3 cells. This suggests that pAKT in GAS6-stimulated G361 cells can recapitulate endogenous pMER activity to a certain degree.

## Conclusions

In summary, pAKT was identified as a robust PD marker and a cellular high-throughput high-content imaging assay was established to assess inhibition of GAS6-induced MER pathway activation. These represent useful tools for successful screening and optimization of small molecules for the treatment of GAS6/MER-driven diseases.

## Data Availability

All data are included in the article.

## Abbreviations

BSA: Bovine serum albumin
DMSO: Dimethyl sulfoxide
DPBS: Dulbecco’s phosphate-buffered saline
ELISA: Enzyme-linked immunosorbent assay
FBS: Fetal bovine serum
FITC: Fluorescein isothiocyanate
GAS6: Growth arrest–specific protein 6
IC_50_: Half-maximal inhibitory concentration
pAKT: Phosphorylated AKT
PD: Pharmacodynamic
pMER: Phosphorylated MER
RPMI: Roswell Park Memorial Institute
siRNA: Small, interfering RNA
TAM: TYRO3/AXL/MER
Trypsin-EDTA: Trypsin-ethylenediaminetetraacetic acid

## References

[1] Lemmon MA, Schlessinger J (2010). Cell signaling by receptor tyrosine kinases. Cell.

[2] Linger RM, Keating AK, Earp HS, Graham DK (2008). TAM receptor tyrosine kinases: biologic functions, signaling, and potential therapeutic targeting in human cancer. Adv Cancer Res.

[3] Lemke G, Rothlin CV (2008). Immunobiology of the TAM receptors. Nat Rev Immunol.

[4] Graham DK, DeRyckere D, Davies KD, Earp HS (2014). The TAM family: phosphatidylserine sensing receptor tyrosine kinases gone awry in cancer. Nat Rev Cancer.

[5] Lee-Sherick AB, Eisenman KM, Sather S, McGranahan A, Armistead PM, McGary CS (2013). Aberrant Mer receptor tyrosine kinase expression contributes to leukemogenesis in acute myeloid leukemia. Oncogene.

[6] Marin-Acevedo JA, Soyano AE, Dholaria B, Knutson KL, Lou Y (2018). Cancer immunotherapy beyond immune checkpoint inhibitors. J Hematol Oncol.

[7] Gan L, Yang Y, Li Q, Feng Y, Liu T, Guo W (2018). Epigenetic regulation of cancer progression by EZH2: from biological insights to therapeutic potential. Biomark Res.

[8] Lemke Greg (2019). How macrophages deal with death. Nature Reviews Immunology.

[9] Sadahiro Hirokazu, Kang Kyung-Don, Gibson Justin T., Minata Mutsuko, Yu Hai, Shi Junfeng, Chhipa Rishi, Chen Zhihong, Lu Songjian, Simoni Yannick, Furuta Takuya, Sabit Hemragul, Zhang Suojun, Bastola Soniya, Yamaguchi Shinobu, Alsheikh Hebaallah, Komarova Svetlana, Wang Jun, Kim Sung-Hak, Hambardzumyan Dolores, Lu Xinghua, Newell Evan W., DasGupta Biplab, Nakada Mitsutoshi, Lee L. James, Nabors Burt, Norian Lyse A., Nakano Ichiro (2018). Activation of the Receptor Tyrosine Kinase AXL Regulates the Immune Microenvironment in Glioblastoma. Cancer Research.

[10] Oien DB, Garay T, Eckstein S, Chien J. Cisplatin and pemetrexed activate AXL and AXL inhibitor BGB324 enhances mesothelioma cell death from chemotherapy. Front Pharmacol. 2017;8:970. 10.3389/fphar.2017.00970 PMC5768913 29375377

[11] Myers Samuel H., Brunton Valerie G., Unciti-Broceta Asier (2015). AXL Inhibitors in Cancer: A Medicinal Chemistry Perspective. Journal of Medicinal Chemistry.

[12] Schlegel J, Sambade MJ, Sather S, Moschos SJ, Tan AC, Winges A (2013). MERTK receptor tyrosine kinase is a therapeutic target in melanoma. J Clin Invest.

[13] Linger RM, Cohen RA, Cummings CT, Sather S, Migdall-Wilson J, Middleton DH (2013). Mer or Axl receptor tyrosine kinase inhibition promotes apoptosis, blocks growth and enhances chemosensitivity of human non-small cell lung cancer. Oncogene.

[14] Shi C, Li X, Wang X, Ding N, Ping L, Shi Y (2018). The proto-oncogene Mer tyrosine kinase is a novel therapeutic target in mantle cell lymphoma. J Hematol Oncol.

[15] Christoph Sandra, DeRyckere Deborah, Schlegel Jennifer, Frazer J. Kimble, Batchelor Lance A., Trakhimets Alesia Y., Sather Susan, Hunter Debra M., Cummings Christopher T., Liu Jing, Yang Chao, Kireev Dmitri, Simpson Catherine, Norris-Drouin Jacqueline, Hull-Ryde Emily A., Janzen William P., Johnson Gary L., Wang Xiaodong, Frye Stephen V., Earp H. Shelton, Graham Douglas K. (2013). UNC569, a Novel Small-Molecule Mer Inhibitor with Efficacy against Acute Lymphoblastic LeukemiaIn VitroandIn Vivo. Molecular Cancer Therapeutics.

[16] Liu J, Zhang W, Stashko MA, Deryckere D, Cummings CT, Hunter D (2013). UNC1062, a new and potent Mer inhibitor. Eur J Med Chem.

[17] Zhang W, DeRyckere D, Hunter D, Liu J, Stashko MA, Minson KA (2014). UNC2025, a potent and orally bioavailable MER/FLT3 dual inhibitor. J Med Chem.

[18] DeRyckere D, Lee-Sherick AB, Huey MG, Hill AA, Tyner JW, Jacobsen KM (2017). UNC2025, a MERTK small-molecule inhibitor, is therapeutically effective alone and in combination with methotrexate in leukemia models. Clin Cancer Res.

[19] Ubil E, Caskey L, Holtzhausen A, Hunter D, Story C, Earp HS (2018). Tumor-secreted Pros1 inhibits macrophage M1 polarization to reduce antitumor immune response. J Clin Invest.

[20] Benzakour O, Gely A, Lara R, Coronas V (2007). Gas-6 and protein S: vitamin K-dependent factors and ligands for the TAM tyrosine kinase receptors family. Med Sci (Paris).

[21] Tang H, Yang J, Shen DR, Calambur D, Witmer M, Wu S (2014). High-throughput high-content imaging assays for identification and characterization of selective AXL pathway inhibitors. Assay Drug Dev Technol.

[22] Massihnia D, Avan A, Funel N, Maftouh M, van Krieken A, Granchi C (2017). Phospho-Akt overexpression is prognostic and can be used to tailor the synergistic interaction of Akt inhibitors with gemcitabine in pancreatic cancer. J Hematol Oncol.

[23] El Bezawy R, De Cesare M, Pennati M, Deraco M, Gandellini P, Zuco V (2017). Antitumor activity of miR-34a in peritoneal mesothelioma relies on c-MET and AXL inhibition: persistent activation of ERK and AKT signaling as a possible cytoprotective mechanism. J Hematol Oncol.

[24] Southworth T, Mason S, Bell A, Ramis I, Calbet M, Domenech A (2018). PI3K, p38 and JAK/STAT signalling in bronchial tissue from patients with asthma following allergen challenge. Biomark Res.

[25] Strickland Amanda L., Rivera Glorimar, Lucas Elena, John George, Cuevas Ileana, Castrillon Diego H. (2019). PI3K Pathway Effectors pAKT and FOXO1 as Novel Markers of Endometrioid Intraepithelial Neoplasia. International Journal of Gynecological Pathology.

